# Dietary Rice Bran-Modified Human Gut Microbial Consortia Confers Protection against Colon Carcinogenesis Following Fecal Transfaunation

**DOI:** 10.3390/biomedicines9020144

**Published:** 2021-02-03

**Authors:** Kristopher D. Parker, Akhilendra K. Maurya, Hend Ibrahim, Sangeeta Rao, Petronella R. Hove, Dileep Kumar, Rama Kant, Bupinder Raina, Rajesh Agarwal, Kristine A. Kuhn, Komal Raina, Elizabeth P. Ryan

**Affiliations:** 1 Department of Environmental and Radiological Health Sciences, Colorado State University, Fort Collins, CO 80523, USA; Kristopher.Parker@frontrange.edu (K.D.P.); hendibrahim1@gmail.com (H.I.); Sangeeta.Rao@colostate.edu (S.R.); 2 Department of Pharmaceutical Sciences, Skaggs School of Pharmacy and Pharmaceutical Sciences, University of Colorado, Anschutz Medical Campus, Aurora, CO 80045, USA; akhilendra28bhu@gmail.com (A.K.M.); dileep07@gmail.com (D.K.); rama.kant@cuanschutz.edu (R.K.); rainabupin@gmail.com (B.R.); rajesh.agarwal@cuanschutz.edu (R.A.); 3 Department of Medical Biochemistry, Faculty of Medicine, Zagazig University, Zagazig 44511, Egypt; 4 Department of Microbiology, Immunology & Pathology, Colorado State University, Fort Collins, CO 80523, USA; Petronella.Hove@colostate.edu; 5 Division of Rheumatology, Department of Medicine, University of Colorado School of Medicine, Anschutz Medical Campus, Aurora, CO 80045, USA; Kristine.Kuhn@cuanschutz.edu; 6 Department of Pharmaceutical Sciences, South Dakota State University, Brookings, SD 57007, USA

**Keywords:** colon cancer carcinogenesis, microbiome, metabolome, fecal microbiota transplantation

## Abstract

Rice bran, removed from whole grain rice for white rice milling, has demonstrated efficacy for the control and suppression of colitis and colon cancer in multiple animal models. Dietary rice bran intake was shown to modify human stool metabolites as a result of modifications to metabolism by gut microbiota. In this study, human stool microbiota from colorectal cancer (CRC) survivors that consumed rice bran daily was examined by fecal microbiota transplantation (FMT) for protection from azoxymethane and dextran sodium sulfate (AOM/DSS) induced colon carcinogenesis in germ-free mice. Mice transfaunated with rice bran-modified microbiota communities (RMC) harbored fewer neoplastic lesions in the colon and displayed distinct enrichment of *Flavonifractor* and *Oscillibacter* associated with colon health, and the depletion of *Parabacteroides distasonis* correlated with increased tumor burden. Two anti-cancer metabolites, myristoylcarnitine and palmitoylcarnitine were increased in the colon of RMC transplanted mice. Trimethylamine-N-oxide (TMAO) and tartarate that are implicated in CRC development were reduced in murine colon tissue after FMT with rice bran-modified human microbiota. Findings from this study show that rice bran modified gut microbiota from humans confers protection from colon carcinogenesis in mice and suggests integrated dietary-FMT intervention strategies should be tested for colorectal cancer control, treatment, and prevention.

## 1. Introduction

The incidence of colorectal cancer is on the rise, today it is the world’s third most deadly cancer with almost 900,000 deaths annually [1] and is expected to increase by 60% to more than 2.2 million new cases and 1.1 million cancer deaths by 2030 [2]. Colorectal cancer is a complex, malignant disease whose multi-stage development involves numerous genetic and environmental risk factors [3]. These risk factors include genetic abnormalities, age, alterations of the gut microbiota [3], and lifestyle-related factors such as smoking, alcohol use, and dietary factors including consumption of highly processed foods, animal fat, and red meat coupled with a low intake of fiber and fruits [4,5]. 

An imbalance in the gut microbiota promotes the progress of colorectal carcinogenesis via multiple mechanisms, including inflammation, activation of carcinogens, and tumorigenic pathways as well as damaging host DNA [6]. Microbial communities involved in the progression and advancement of colorectal cancer have been reviewed globally [3,7], with various studies showing microbiota connections to the development of gastrointestinal cancers [8,9]. In germ-free mice, lower incidence of chemically induced duodenal and colonic tumors were observed compared to those raised in conventional husbandry [10]. Additionally, bacterial involvement in carcinogenesis was supported by studies that detected a reduction of tumor load after antibiotic manipulation of gut microbiota [11,12]. Fecal microbiota transplantation (FMT) from healthy donors and adults with CRC into germ-free mice treated with azoxymethane and dextran sodium sulfate (AOM/DSS) identified a number of microbial signatures associated with tumorigenesis [13]. A similar study using AOM alone [14], showed enrichments of *Bacteroides fragilis* and depletions of *Faecalibacterium prausnitzii* in germ-free mice transfaunated with CRC-associated stool. There is evidence that *Akkermansia* and taxa in the order Bacteroidales (e.g., *Alistipes*, *Bacteroides*, and *Parabacteroides*) were positively associated with higher rates of tumorigenesis and enrichments of *Roseburia*, *Blautia*, and *Subdoligranulum* were reported in mice with a higher tumor burden. On the other hand, there is evidence of anti-cancer effect of certain microbial communities. For instance, Clostridiales-related taxa, including *Lachnospiraceae* and *Clostridium* Group XIVa were associated with decreased tumor burden [13], implying the potential benefits of transplanting beneficial microbiota in the treatment and control of CRC. However, studies regarding fecal microbiota transplantation in colorectal cancer patients remain limited [6]. This underscores the importance of further investigating the role of FMT to prevent, treat, and control disease progression via modifications to intestinal microbial communities [15]. 

In addition, while dietary intervention has been shown to reduce disease recurrence and mortality [16,17,18], the elevated intake of whole grains merits specific attention for modulation of gut microbial communities that mitigate multiple hallmark cancer mechanisms [19,20]. Rice bran, an agricultural byproduct of white rice milling, has demonstrated efficacy for the control and suppression of CRC in carcinogen treated rats and mice [21]. Rice bran contains nutrients, vitamins, phytosterols, fatty acids, polyphenols, prebiotics, and other phytochemicals that have antioxidant, immunomodulatory, mucosal protective, and anti-cancer activities [22,23,24]. Short term feeding trials with rice bran in CRC survivors showed production of metabolites in the stool, blood, and urine [25,26]. Changes in gut microbiota metabolism were assessed in humans following dietary intake of rice bran in adults [16,25]. Microbial metabolites [27] produced by bacterial fermentation of undigested dietary components that reach the large intestine connect intestinal microbiota to CRC [28]. The short-chain fatty acid (SCFA), butyrate, derived from fermentation of fiber has been shown to play a role in the suppression of CRC [29] and trimethylamine N-oxide (TMAO), a gut microbial metabolite of dietary meat, has shown a strong link with colorectal cancer [30].

This study highlights that nutritional intervention with rice bran modifies microbiota for prevention and control of CRC and has potential to reduce disease recurrence by improving metabolism by gut microbiota [31,32] alongside opportunities for fecal microbiota transplantation (FMT). In the present study, we first tested the effectiveness of rice bran-modified human gut microbiota to reduce colon tumor formation via FMT in AOM/DSS treated mice. The hypothesis was that rice bran-modified human gut microbiota (RMC) produces metabolites that reduce colon inflammation and inhibit growth and progression of carcinogen-induced neoplastic lesions in the murine colon.

## 2. Materials and Methods

Ethics. Stool samples were collected following written informed consent under the approved human subjects’ protocol and registered clinical trial (ClinicalTrials.gov Identifier: NCT01929122) and findings from this human clinical study with rice bran were previously published [16,33]. Animal experiments were performed under institutional guidelines using approved Institutional Animal Care and Use Committee (IACUC) protocol [(B-57916(04)1E] in the gnotobiotic core facility at UC Denver-AMC. 

Human donor characteristics and stool microbiota inoculum preparation. Stool microbial consortia from CRC survivors enrolled in the randomized controlled trial (NCT01929122) were selected as microbiota inoculums for FMT into germ-free mice. Information regarding recruitment, exclusion criteria, design, and sampling in the CRC survivors’ trial has been described in detail previously [33]. Stool from a 68-year old female CRC survivor before and after 30 g/day of rice bran intake for 28 days represented the control microbiota consortium (CMC-f) and rice bran modified microbiota consortium (RMC-f), respectively. Stool microbiota from the 67-year old male without any dietary intervention represented the control microbiota consortium (CMC-m) and a sample from the 51-year old male after 30 g/day of rice bran intake for 28 days represented the rice bran modified microbiota consortium (RMC-m). Inoculums were prepared using a 1:1 ratio of fresh stool to PBS + 60% glycerol (0.25 g of stool was used for each preparation). Human stool inoculums were stored at −80 °C until used for murine transplantation. Glycerol was removed prior to oral gavage of inoculums into mice. Briefly, inoculum samples were thawed and washed with at least 10-fold volumes of PBS, centrifuged at 2000 rpm for 10 min at 4 °C, and then diluted to the original volume with PBS. For transplantation, 200 μL was used to orally gavage the germ-free mice.

Animal facility procedures and treatments. C57BL/6 germ free mice breeding colony and germ-free litters were housed in sterile vinyl isolators in the gnotobiotic facility. Only trained staff (specifically trained in germ-free animal care to minimize possible cross contamination of isolators) worked on the fecal microbiota transplantation (FMT) and related procedures. Throughout the duration of the study, mice were either maintained in isolators in gnotobiotic facility or moved to autoclaved static cages (upon colonization and exposure to carcinogen) and provided with ad libitum access to autoclaved water and gamma-irradiated (suitable for germ-free facility) AIN-93M pellet diet (Envigo). 

Germ free mice aged 8–10 weeks and weighing ~20 to 25 g were randomized into five groups according to treatment: vehicle/no inoculum, N (*n* = 3); CMC-f (*n* = 7) mice that received microbiota from control female inoculum prior to rice bran dietary intervention; RMC-f (*n* = 9) mice that received rice bran modified microbiota consortium. The CMC-m (*n* = 9) mice received gut microbiota consortium from control male prior to rice bran dietary intervention and RMC-m (*n* = 10) mice received rice bran modified microbiota consortium. Mice in each group were orally gavaged with 200 μL of the prepared human stool inoculum on the first day and then again after 4 days. Fourteen days after the first gavage, gut microbiota colonization was confirmed through testing of murine fecal samples by qPCR. Following confirmation of microbiota colonization (21 days after the first gavage), all mice received a single intraperitoneal (i.p) injection of 10 mg/kg AOM (Sigma Aldrich) in saline. Seven days after AOM injection, mice were exposed to 2% DSS (DSS-mol.wt 36,000–50,000, MP Biomedicals, LLC, Santa Ana, CA, USA) in drinking water for five days with one water change once after 2 days providing fresh DSS). This was then followed by 14 days of normal drinking water (resting phase). This cycle (DSS exposure + resting phase) was repeated twice to yield a total of three DSS cycles. Body weight, food consumption, and general health (stool consistency, incidents of bloody diarrhea/loose stools, and rectal-prolapse were monitored closely for confirmation of colitis events) of mice was recorded weekly over the course of the study. In addition, fecal samples/cage were collected as in all control and rice bran microbiota treated groups at the following points: before AOM, before DSS cycle 1; before DSS cycle 2; before DSS cycle 3; four-weeks after DSS cycle 3; and at the end of the study (14 weeks post-AOM), and immediately stored frozen at −80 °C for DNA extraction. 

At the end of study, mice were subjected to CO_2_ asphyxiation and euthanized by exsanguination. Blood was collected in BD vacutainer K2 EDTA coated tubes followed by centrifugation for 15 min at 2000× *g* at 4 °C resulting plasma was stored at −80 °C until ready for metabolomics. Following euthanizer, the cecum and its contents were collected, snap frozen in liquid nitrogen, and stored at −80 °C until processed for metataxonomics. The entire colon was excised starting from the cecum to the distal end and cut open longitudinally along its main axis for metataxonomics and lesion evaluation. Swabs of colon contents from the initial part of proximal, and lower part of distal colon or dirty segments of associated colon tissue (~1 mm) were collected and stored at −80 °C for metataxonomics (16S rRNA). Next, colon tissue was gently flushed with ice cold saline solution and cleaned with a fine brush to remove remnant luminal contents; colon was viewed under a dissecting microscope to count the number of neoplastic lesions in addition to measuring the diameter of lesions using digital calipers. Following this, ~1 to 2 mm slivers (horizontal) of clean colon tissue from the proximal and distal ends were cut, snap frozen in liquid nitrogen, and stored at −80 °C until metabolomics processing. 

Metataxonomics sample processing, sequencing, and analysis. Thawed cecum contents, dirty colon tissue, and fecal samples were homogenized (separately) prior to DNA extraction with the MoBio PowerSoil Kit (MoBio Laboratories Inc., Carlsbad, CA, USA) per manufacturer protocols. The V4 hypervariable region of the 16S rRNA gene was amplified and sequenced on the Illumina MiSeq platform according to the Earth Microbiome Project [34] standards using the 515F Parada [35] and 806R Aprill [36] primer pair. A total of 3,464,689 raw single-end FASTQ formatted forward sequence reads represented by 147 samples were imported into the Quantitative Insights into Microbial Ecology 2 (QIIME 2) [37]. A feature table comprised of amplicon sequence variant (ASV) absolute abundances for each sample was inferred from reads using the Divisive Amplicon Denoising Algorithm 2 (DADA2) pipeline [38]. Taxonomic identities for ASV representative sequences were assigned with Naïve Bayes classifiers independently trained on 99% OTU reference collections bound by the 515F/806R (Parada/Aprill) primer pair and trimmed to 248 bp extracted from either Greengenes 13_8 [39] or SILVA 132 [40] marker gene databases. The raw ASV table, representative sequences, and taxonomy tables were exported from QIIME 2 for further processing in R. A master table comprised of ASV representative sequences, full and truncated taxonomic lineages, and raw absolute abundances for all ASVs within all samples was constructed using base R in combination with package dplyr. Potential contaminant ASVs assigned by either database as chloroplast, mitochondria, eukaryote, or unassigned kingdom were removed from the master table. Any samples exhibiting excess of 1% relative abundance of contaminants and any biological samples with total absolute ASV abundance fewer than 999 were removed from the master table. Samples analyzed after processing included 142 biological samples represented by 17 human stool microbiota inoculums, 27 murine cecum, 49 murine colon (25 proximal; 24 distal), and 49 murine fecal samples. A previously described approach [38] was followed to construct a midpoint rooted phylogenetic tree from ASV representative sequences using R packages Biostrings, DECIPHER, phangorn, and ape. The phylogenetic relatedness of microbial communities were compared using three UniFrac distance metrics: generalized, unweighted, and weighted [41]. UniFrac distance matrices were computed using R package GUniFrac and ordinated with principal coordinates analysis (PCoA) using R packages ape, dplyr, ggplot2, and ggpubr. Comparisons of phylogenetic-independent microbiota composition (i.e., composition of ASVs) proceeded using the compositional data analysis paradigm [42]. Zero counts for ASVs were imputed using the count zero multiplicative (CZM) method from R package zCompositions followed by applying the centered log-ratio (clr) transformation with log base 2. The relationships between samples were visualized through principal components analysis (PCA) and unsupervised hierarchical clustering via Ward’s method [43] with multiscale bootstrap resampling (*n* = 10,000). Dendrograms were constructed using R packages dendextend, dplyr, ggplot2, ggpubr, ggraph, and pvclust while packages dplyr, ggbiplot, ggplot2, and ggpubr were used for PCA illustration. For ease of interpretation, taxonomic composition at the phylum, family, and lowest possible assignment levels were depicted as proportions (relative abundances) with plots constructed using packages dplyr, ggplot2, ggpubr, and reshape2. Differential abundance testing at the phylum, family, lowest assignment, and ASV levels was conducted with ALDEx2 [44] from Bioconductor suite [45]. For a given differentially abundant ASV or taxon, log_2_ fold differences between groups were visualized using packages dplyr, ggplot2, and ggpubr. For full details regarding metataxonomics protocols and analysis, including PCR conditions, product purification, library pooling, primer sequences, ASV feature table construction, R package citations, and additional computational information see Supplementary Methods.

Non-targeted metabolomics: sample processing, metabolite identification and analysis. Clean (devoid of any colon fecal contents) proximal and distal colon tissue (50 mg/sample) and plasma (1 mL/sample) from mice were sent on dry ice to Metabolon Inc^©^ for metabolite extraction and identification as previously described [46]. An internal database comprised of more than 3300 commercially available chemical standards was utilized for compound identification. Annotations of compounds were made based upon matches in retention time/index, possessing a mass to charge ratio (*m*/*z*) within 10 parts per million, and assessment of overall mass spectral profile. Spectral profiles that were structurally resolved but were not archived in the internal database were reported as unknown. The complete description of sample processing and metabolite identification can also be found in the Supplementary Materials. Metabolite abundances were median scale normalized by dividing the raw abundance of a metabolite by the median raw abundance of that metabolite across the entire dataset. Median scaling was performed individually for each matrix colon. For samples lacking a metabolite, the minimum median-scaled abundance of that metabolite across the given dataset was used as the input value. Metabolite composition explored through PCA ordination followed the compositional data analysis framework described above for metataxonomic data analysis. For a given differentially abundant metabolite, fold differences between groups were transformed using log base 2 prior to visualization using packages dplyr, ggplot2, and ggpubr.

Statistical Analysis. The non-parametric Kruskal–Wallis test was utilized to determine differences in lesion number across consortium groups matched by sex of human donor. Pairwise comparisons were performed using the non-parametric Wilcoxon rank-sum test with the resultant *p*-values adjusted for multiple comparisons using the Benjamani–Hochberg (BH) procedure [47]. Non-parametric testing was performed after the results of Shapiro–Wilk normality testing indicated non-normal distributions. Non-parametric permutational ANOVA from the R package vegan was utilized to detect differences between groups in phylogenetic relatedness of microbial communities with UniFrac distance matrices as inputs and ASV composition using the Aitchison distance metric. ALDEx2 testing was performed as follows: 1000 Monte Carlo (MC) instances of the Dirichlet distribution for each sample were generated from the respective subset tables containing absolute abundance data; the clr transformation was then applied over each MC instance; *p*-values were produced using the non-parametric Wilcoxon rank-sum test, to compare each ASV/taxon’s clr abundance values between the specified two groups; *p*-values were adjusted for multiple comparisons using the Benjamani–Hochberg (BH) procedure [47] resulting in adjusted *p*-values (henceforth referred to as BH-*p*); *p*-values and BH-*p*-values for each ASV/taxon were averaged across all MC instances to yield expected *p*-values and expected BH-*P*-values. Any ASV/taxon with an expected BH-*p*-value less than 0.1 was deemed significant. The package BiocParallel from the Bioconductor suite was used to execute ALDEx2 functions using multi-core processing to drastically reduce computational time. Differential abundance testing of metabolites between groups was carried out using two-way ANOVA and Welch’s post-hoc tests. Any metabolite with a *p*-value less than 0.05 was deemed significant. A *q*-value was calculated for each metabolite to account for false discovery rate errors.

## 3. Results

### 3.1. Rice Bran-Modified Human Fecal Microbiota Transplant (FMT) Elicited Protection from AOM/DSS-Induced Colon Carcinogenesis

**Figure 1:**
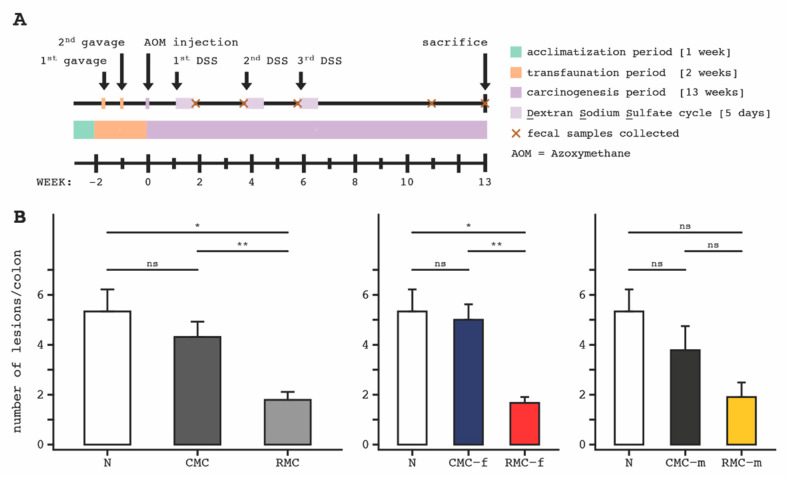
Study design and colon tumor outcomes following human fecal microbiota transplantation (FMT) in AOM-DSS treated mice. (**A**), Timeline for FMT gavage and AOM-DSS treatments to male and female mice. Murine feces was collected over time and tissues were harvested at 13 weeks for microbiota and metabolomics endpoint analyses. (**B**), Number of neoplastic lesions in entire colon tissue for mice receiving no microbial consortia, N (*n* = 3), when compared to control microbial consortia (CMC) and rice bran modified microbial consortia (RMC) from human male (m) and female (f). The control (*n* = 16 mice) and rice bran (*n* = 19 mice) for both sexes combined for the human microbiota donors (left). The no treatment N (*n* = 3); control microbiota consortia from female, CMC-f (*n* = 7) and rice bran modified microbiota consortia from female, RMC-f (*n* = 9) (middle). 68.2% reduction in number of lesions for RMC-f compared to CMC-f. The no treatment, N (*n* = 3); control microbiota consortia from male, CMC-m (*n* = 9); rice bran modified microbiota consortia from male, RMC-m (*n* = 10). 49.7% reduction in number of lesions for RMC-m compared to CMC-m (right). Error bars represent standard error of the mean; (*) indicates Benjamani–Hochberg adjusted *p*-value < 0.05; (**) indicates Benjamani–Hochberg adjusted *p*-value < 0.01; (ns) indicates Benjamani–Hochberg adjusted *p*-value > 0.05.

Human stool microbiota from female and male CRC survivors taken before and after completing a dietary rice bran intervention for 28 days were evaluated for the functional capacity to reduce chemically induced colon carcinogenesis in germ-free mice. The study design, timeline of murine FMT, microbiota colonization, AOM treatment, cycles of DSS, and murine biospecimen collection are depicted in (Figure 1A). 

Colon tissue of mice transplanted with the rice bran-modified microbiota consortium (RMC) displayed significantly fewer neoplastic lesions compared to mice that received the control microbial consortium (CMC), and those receiving no inoculum (N) (Figure 1B, left). Effects of human RMC-FMT on murine tumor reduction by sex of the donor is also shown (Figure 1B, middle and right). Total neoplastic lesions of mice receiving rice bran-modified microbiota from the human female donor (RMC-f) showed a significant decrease in lesions when compared to control (CMC-f), Benjamani–Hochberg adjusted *p*-value [47] (*BH-p* = 0.003) and from mice receiving no inoculum (N) (*BH-p* = 0.019) (Figure 1B middle). The distal colon showed the greatest reduction in lesions (68.2%) (*BH-p* = 0.022) (Supplementary Table S1 and Figure S1A). A 49.7% reduction was observed in the total number of colon lesions from mice transplanted with rice bran modified microbiota from the human male donor (RMC-m) when compared to the control (CMC-m) and no inoculum (Figure 1B, right). Importantly, these effects were independent of murine sex (Supplementary Figure S1C,D).

### 3.2. Metataxonomics of Human Stool Microbiota Inoculums Used for Fecal Microbiota Transplantation (FMT) in AOM-DSS Treated Germ-Free Mice

Metataxonomics (16S rRNA gene sequencing) was used to elucidate the composition of gut microbiota stool inoculums collected from CRC survivors before and after dietary rice bran intake for one month. In addition to the classic compositional nature of microbiome datasets acquired from next-generation sequencing platforms, we also compared the non-phylogenetic community composition using the Aitchison distance metric with centered log-ratio transformed abundance data [42]. We computed three phylogenetic-based beta diversity metrics (generalized, unweighted, and weighted UniFracs) and compared inoculum groups using pairwise PERMANOVA. The human female (RMC-f) that differentially reduce colon tumors in mice and from control (Figure 1B) did not exhibit statistical differences in beta diversity (Supplementary Table S1). Statistical differences were demonstrated between stool inoculums derived from human males for generalized (*p* = 0.009) and unweighted UniFracs (*p* = 0.007) and the Aitchison distance metric (*p* = 0.009), while no difference was observed for weighted UniFrac (*p* = 0.084). 

Next, we utilized the ALDEx2 R package to assess for differentially abundant taxa at the phylum, family, and lowest possible assignment levels between the control and rice bran modified human stool inoculums matched by the sex of human donor. A total of 28 taxa represented by 1 phylum, 9 families, 14 genera, and 4 species were enriched in the female rice bran—modified inoculum, and a total of 19 taxa represented by 2 families, 14 genera, and 3 species were enriched in the male control inoculum (Supplementary Table S1). The rice bran modified inoculums exhibited enrichments of *Akkermansia*, *Bacteroides uniformis*, *Blautia hydrogenotrophica*, *Coprococcus*, *Collinsella*, and Lachnospiraceae. Taxa enriched in the control inoculums included *Bacteroides fragilis*, *Blautia*, *Enterococcus*, *Erysipelatoclostridium*, *Faecalibacterium*, and *Parabacteroides distasonis*. 

After assessing for differentially abundant taxa, we then performed differential abundance testing at the amplicon sequence variant (ASV) level which identified 36 ASVs enriched in the rice bran modified inoculum and 18 ASVs enriched in the control inoculum. As expected, taxonomic assignments for differentially abundant ASVs shared considerable overlap when compared to the results obtained from testing at explicit taxonomic ranks; however, several key differences were observed. These differences included ASVs assigned to the genera *Alistipes*, *Dorea*, *Faecalibacterium*, and *Roseburia* enriched in the rice bran modified inoculum and ASV affiliated with the genus *Anaerostipes* enriched in the control inoculum. Supplementary Table S2 lists the differentially abundant taxa and ASVs in human stool microbiota used as inoculums and associated murine colonization in cecum, proximal colon, and distal colon after FMT. There was no statistical support for differentially abundant taxa or ASVs in the consortia derived from the human female as was shown with the male. 

### 3.3. Human Stool Microbiota Comparisons after Transplantation and across Murine Cecum, Proximal Colon, and Distal Colon

**Figure 2:**
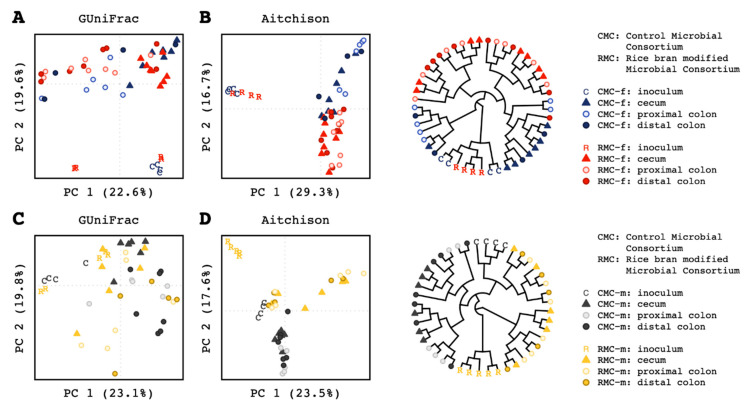
Human control and rice bran modified microbial consortia (CMC and RMC inoculum) compared to murine cecum and colon (proximal and distal) microbiota composition. (**A**) Principal Coordinates Analysis of generalized UniFrac distances for mice colonized with consortia derived from the human female. (**B**)Principal Components Analysis (left) and Ward D2 hierarchical clustering (right) of Aitchison distances for mice colonized with consortia derived from the human female. (**C**) Principal Coordinates Analysis of generalized UniFrac distances for mice colonized with consortia derived from the human males (CMC-m/RMC-m). (**D**) Principal Components Analysis (left) and Ward D2 hierarchical clustering (right) of Aitchison distances for mice colonized with consortia derived from the human male. (**A**–**D**)letters indicate human stool inoculum samples, shapes distinguish murine sample type, and colors denote sex of donor (see key for female: red/blue and male: yellow/black).

Given the human female donor stool (RMC-f) resulted in significantly decreased number of neoplastic lesions in the colon after transplantation that was not associated with diversity, the targeted investigation of microbiota did show changes in composition across the murine cecum, colon and feces. Pairwise PERMANOVA comparisons for the mice groups receiving control and rice bran modified microbiota consortium showed differences in generalized (*BH-p* = 0.007), unweighted (*BH-p* = 0.005), and weighted (*BH-p* = 0.018) UniFracs and Aitchison distance for the cecum (*BH-p* = 0.001); differences in generalized (*BH-p* = 0.003) and weighted UniFracs (*BH-p* = 0.007) and Aitchison distance (*BH-p* = 0.001) for the proximal colon; and differences in Aitchison distance (*BH-p* = 0.02) for the distal colon. Within group comparisons by murine tissue and by location indicated statistical support for differences in microbial community composition along the intestinal tract (Supplementary Table S1). Tissue location-based differences were largest between the cecum and proximal colon communities, while the proximal and distal colon locations harbored similar compositions across all four metrics analyzed. Principal coordinates analysis (PCoA) of generalized UniFrac distances visually demonstrated the differences in sampling location within groups (Figure 2A); differences between groups for the same tissue type were also apparent. Visualization of Aitchison distances using principal components analysis (PCA) and hierarchical clustering corroborated the results of PERMANOVA while further highlighting the compositional differences between consortium groups (Figure 2B). 

For all murine tissue with control microbiota consortia (CMC) and rice bran modified microbiota (RMC), PERMANOVA indicated statistical support for differences in Aitchison distance (*BH-p* = 0.005), but not for any of the UniFrac metrics (Supplementary Table S1). These results were corroborated when PCoA of generalized UniFrac along with PCA and hierarchical clustering of Aitchison distances were visualized (Figure 2C,D). 

### 3.4. Taxonomic Comparisons of Mouse Intestinal Microbiota by Sex of the Human Donor Fecal Microbiota Inoculum Used for Transfaunation

Differences in the microbial community composition along the murine intestinal tract shown in Figure 2. prompted the comparative investigations of taxonomic composition in murine cecum, proximal colon, distal colon, and feces (control and rice bran modified group) by sex of the human donor. 

**Figure 3:**
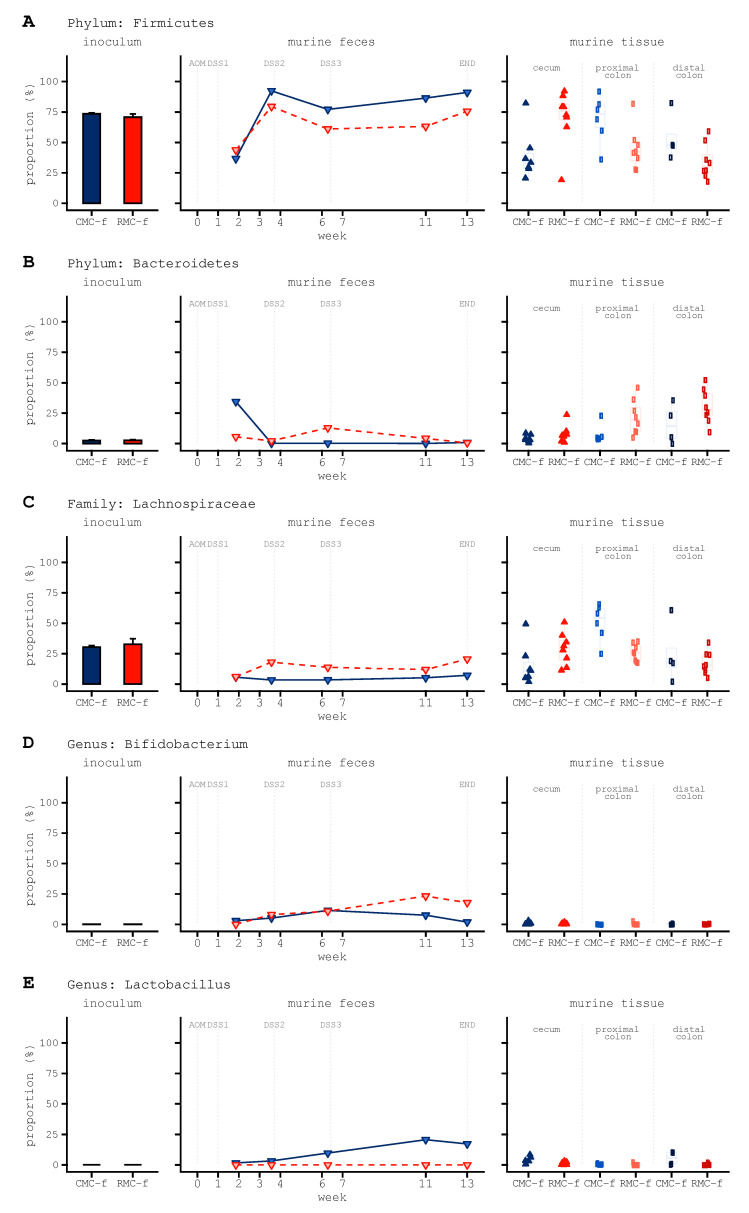
Taxonomic composition (proportions) of human female donor stool inoculums into murine feces, cecum, and colon. (**A**) Firmicutes (**B**) Bacteroidetes (**C**) Lachnospiraceae. (**D**) *Bifidobacterium* (**E**) *Lactobacillus* (**A**–**E**) the y-axis is identical across plots within a given panel and is set at 100% for all panels depicted. Taxonomy was collapsed at the phylum (**A**,**B**) family (**C**) or genus- (**D**,**E**) levels to combine proportions for identical assignments prior to visualization. Taxonomy was assigned using the SILVA 132 marker gene database.

Murine tissue specific differences from receiving human female donor microbiota consortium (RMC-f) included decreased abundance for the phylum Firmicutes in the murine cecum, the proximal and distal colons, and feces. (Figure 3A). Firmicutes that dominated the cecum of mice included the family Lachnospiraceae (Figure 3C), although a lower proportion of Lachnospiraceae was observed in the proximal colon. In contrast to the trend observed for Firmicutes, mice receiving RMC-f exhibited higher abundance of Bacteroidetes across sample locations (Figure 3B). Because rice bran was previously reported to enrich native probiotic bacteria in human stool [26], we next took a targeted approach to explore taxa belonging to the genera *Bifidobacterium* and *Lactobacillus*. This analysis revealed increased *Bifidobacterium* in the feces of mice receiving rice bran modified consortium (Figure 3D) and decreased *Lactobacillus* in the cecum and distal colon (Figure 3E).

Next, comparisons were made on murine tissue and feces using a targeted approach that explores the abundances of a variety of taxa previously associated with CRC [3] or colon tumor burden [13]. The series of taxa previously reported with associations with colorectal cancer and colon tumors are listed in Supplementary Table S3.

- Genus *Akkermansia* exhibited higher abundance in the rice bran modified microbiota (RMC inoculum), and there were similar abundances and changes over time in the feces of mice. There was substantially higher *Akkermansia* abundance in the cecum of mice receiving control inoculum; and qualitatively higher abundance in the proximal and distal colon of mice receiving the rice bran modified inoculum (Supplementary Figure S2A, left).
- Genus *Alistipes*, *Bacteroides*, and *Bacteroides uniformis* were more abundant in the cecum and both colon locations while minimally abundant in the human donor inoculums and murine feces (Supplementary Figure S2B,C,J, left).
- The rice bran modified microbiota donor inoculum and the murine feces contained a higher relative abundance of *Blautia*; however, the cecum, proximal colon, and distal colon of these showed lower abundance of *Blautia* when compared to the control group (Supplementary Figure S2D, left).
- Striking reductions of *Escherichia*–*Shigella* and *Parabacteroides* were observed between the first and second fecal collection timepoints, with the former taxon relatively non-existent in the murine gut while *Parabacteroides* was more abundant across the cecum, proximal, and distal colon of mice receiving control inoculum (Supplementary Figure S2E,F, left).
- *Bacteroides fragilis* was relatively absent in most gut tissues of FMT mice with the exception of the distal colon of mice receiving rice bran modified microbiota (RMC) inoculum (Supplementary Figure S2G, left).
- The control inoculum and the proximal colon from mice transfaunated with this inoculum displayed higher abundance of *Roseburia*, while minimal differences were seen in the murine cecum or the distal colon in both control and rice modified inoculum groups (Supplementary Figure S2H, left); *Roseburia* showed near zero abundance in murine feces.
- Each of the tissue samples from mice receiving the rice modified microbiota donor inoculum harbored higher abundance of a *Ruminococcus* taxon with a contested assignment (Supplementary Figure S2I, left).
- *Anaerostipes*, *Faecalibacterium*, and *Ruminococcus* were observed in the human donor inoculums for FMT, yet these taxa did not appear to effectively colonize the murine gut (Supplementary Figure S2K–M, left).
- ALDEx2 differential abundance testing identified two genera and three ASVs with affiliations to *Flavonifractor* and *Holdemania* enriched in the cecum samples of RMC-f transfaunated mice, while cecum samples from CMC transfaunated mice were distinguished by an increase in *Parabacteroides distasonis* (Figure 3A and Supplementary Table S2).

**Figure 4:**
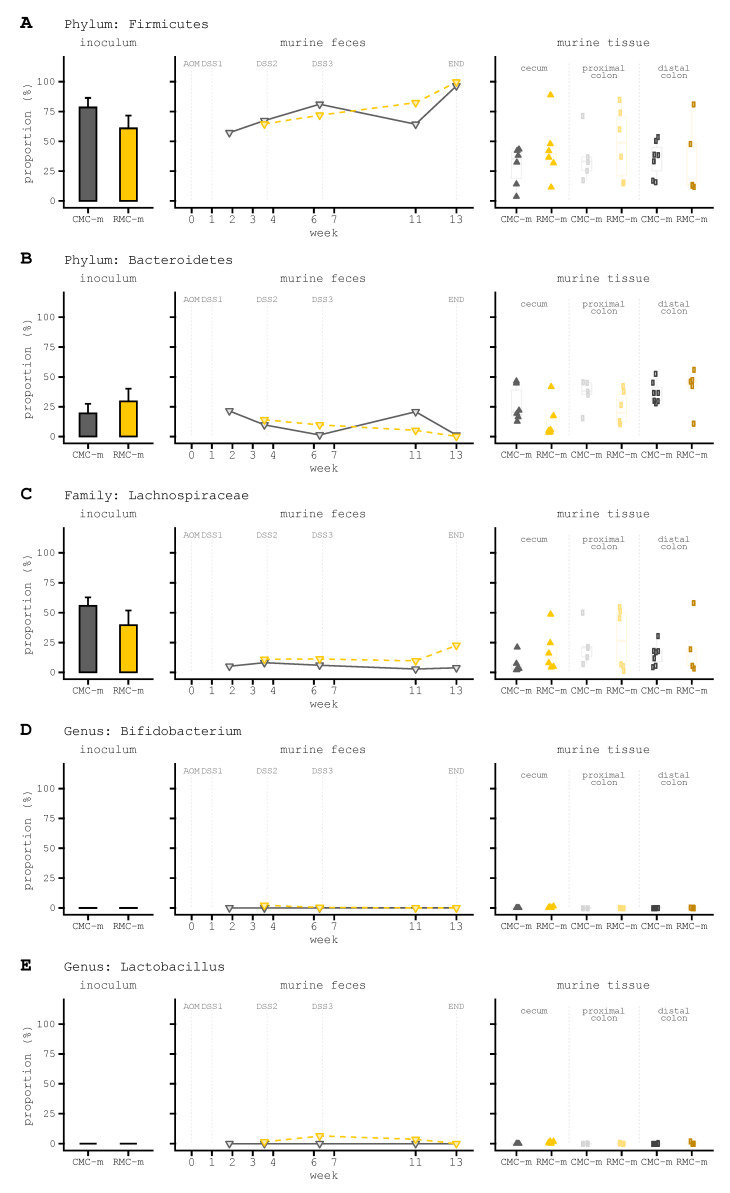
Taxonomic composition (proportions) of human male donor stool inoculums into murine feces, cecum, and colon. (**A**) Firmicutes proportions for stool inoculums and samples from mice colonized with consortia derived from the human male. (**B**) Bacteroidetes (**C**) Lachnospiraceae (**D**) *Bifidobacterium* (**E**) *Lactobacillus* (**A**–**E**) the y-axis is identical across plots within a given panel and is set at 100% for all panels depicted. Taxonomy was collapsed at the phylum (**A**,**B**) family (**C**) or genus- (**D**,**E**) levels to combine proportions for identical assignments prior to visualization. Taxonomy was assigned using the SILVA 132 marker gene database.

Murine tissue specific differences after transplantation with human male donor microbiota consortium involved Firmicutes and Lachnospiraceae with higher abundances across cecum, proximal colon, distal colon, and later fecal timepoints in mice even with these taxa showing lower abundance in the donor inoculum (Figure 4A,C).

- An opposite trend was observed for Bacteroidetes as this phylum displayed higher abundance in RMC-m, with lower abundance in each of the murine tissues and several of the fecal timepoints (Figure 4B, right).
- Both *Bifidobacterium* and *Lactobacillus* had minimal abundance (Figure 4D,E), with the exception of two fecal timepoints showing a higher abundance of *Lactobacillus* in mice receiving rice bran modified inoculum (Figure 4E, middle).
- The targeted analysis of taxa indicated lower abundances of *Akkermansia, Alistipes, Roseburia,* and *Bacteroides uniformis* mice despite higher abundance in the rice bran modified inoculum (Supplementary Figure S2A,B,H,J, right). The opposite relationship was observed for *Blautia*; (Supplementary Figure S2D, right).
- Higher abundances of *Bacteroides, Escherichia*–*Shigella*, and *Ruminococcus* were detected in the rice bran modified inoculum and tissue samples from mice receiving this (Supplementary Figure S2C,E,I, right).
- *Parabacteroides* and *Bacteroides fragilis* were lower in rice bran modified inoculums and corresponding mice samples (Supplementary Figure S2F,G, right). Despite relatively high proportions in the inoculums, *Anaerostipes*, *Faecalibacterium*, and *Ruminococcus* also exhibited poor colonization in the mice receiving both rice bran modified and control inoculum (Supplementary Figure S2K–M, right).

#### Integrated Summary of Intestinal Tissue Differences Observed for Human Female and Male Rice Bran Modified Microbiota (RMC) Transplantation

**Figure 5:**
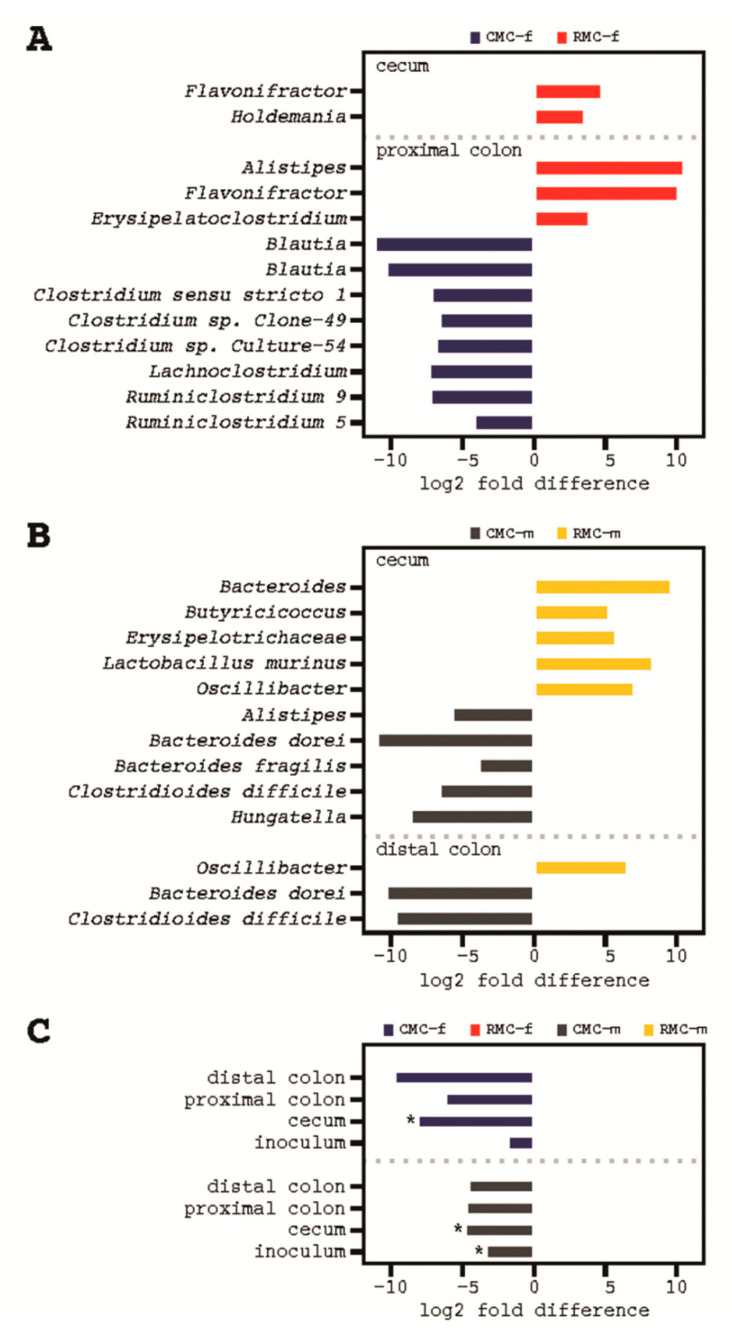
Differentially abundant taxa in murine tissue and feces following transplantation with control human inoculum (CMC) and rice bran modified human inoculum (RMC) treatments. (**A**), taxa in mice transfaunated with consortia from the human female donor. (**B**), taxa in mice transfaunated with consortia from the human male donors. (**C**), The one differentially abundant taxon (*Parabacteroides distasonis)* with conserved depletion in mice transfaunated with RMC from the human female and male donor; asterisks indicate sample type with *BH-p* < 0.1. Differential abundance testing was performed using ALDEx2 with *n* = 1000 Monte Carlo simulations; Wilcoxon rank-sum testing and false discovery rate correction using the Benjamini–Hochberg procedure. Taxonomy was assigned using the SILVA 132 marker gene database.

For the mice receiving rice bran modified female inoculum proximal colon samples showed enrichments of *Flavonifractor* along with ASVs affiliated with the genera *Alistipes* and *Erysipelatoclostridium* and depletions of *Blautia*, *Clostridium* spp., *Lachnoclostridium*, and *Ruminiclostridium*. (Figure 5A and Supplementary Table S2). No statistical support for differentially abundant ASVs or taxa was observed in the distal colon of rice bran modified or control consortium in mice.

For the mice receiving with rice bran modified male inoculum ALDEx2 differential abundance testing indicated enrichments of *Oscillibacter* in both the cecum and distal colon, in addition to enrichments of *Bacteroides Butyricicoccus*, *Erysipelotrichaceae*, and *Lactobacillus murinis* in the cecum (Figure 5B and Supplementary Table S2). Mice transfaunated with control inoculum were distinguished by a variety of taxa including affiliations with *Alistipes*, *Bacteroides fragilis*, *Bacteroides dorei*, *Clostridioides difficile*, *Hungatella*, and *Parabacteroides distasonis*, all enriched in the cecum with *B. dorei* and *C. difficile* also enriched in the distal colon (Figure 5B and Supplementary Table S2). No statistical support for differentially abundant ASVs or taxa was observed in the proximal colon of mice transfaunated with consortia from any of the human male donors.

To understand microbiota potentially responsible for conferring protection or implicated in tumor development, we compared sets of differentially abundant taxa and ASVs across consortium groups. We sought to determine any ASV that was enriched in all samples from both RMC-f and RMC-m. This analysis identified a conserved depletion of the family Tannerellaceae and a lower-level assignment within this family, *Parabacteroides distasonis*, in the inoculums from the female and male donors, the cecum, and the proximal and distal colon regions of the RMC-transfaunated groups (Table 1 and Figure 5C).

### 3.5. Human Donor Stool Inoculum Altered Murine Blood and Tissue Metabolite Profiles and Revealed FMT-Differences in Gut Microbiota Metabolism

#### 3.5.1. Murine Metabolite Changes from Human Female FMT before and after Rice Bran Intervention

**Figure 6:**
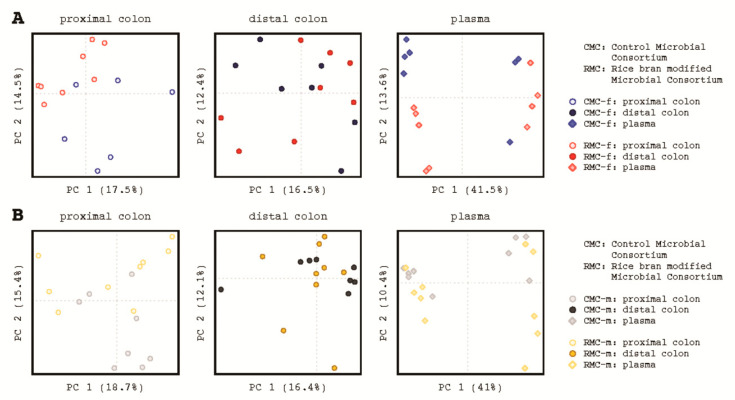
Principal Components Analysis of murine colon tissue (proximal and distal) and plasma metabolome. (**A**), Aitchison distances for proximal colon tissue (left), distal colon tissue (middle), and plasma (right) from mice colonized with consortia derived from the human female (**B**), Aitchison distances for proximal colon tissue (left), distal colon tissue (middle), and plasma (right) from mice colonized with consortia derived from the human males. Shapes distinguish murine sample type and colors denote male or female donor control or rice bran group.

Murine transplantation with rice bran modified inoculum from both male and female donors elicited several consistent metabolite changes. The metabolite composition of murine tissues and plasma from following CMC-f and RMC-f were visualized using PCA and indicated distinct separation for proximal colon tissue (Figure 6A, left), minimal separation of distal colon tissue (Figure 6A, middle), and clear separation of murine plasma (Figure 6A, right). 

Differential abundance testing of colon tissue metabolites between mice receiving RMC-f and CMC-f identified a total of 62 differentially abundant compounds in the proximal colon (50 increased; 12 decreased in those receiving rice bran modified inoculum), 24 compounds in the distal colon (7 increased; 17 decreased in in those receiving rice bran modified inoculum), and 169 compounds in the plasma (90 increased; 79 decreased in in those receiving rice bran modified inoculum) (Supplementary Table S4). Across both colon tissue locations, two identified metabolites (*N*6-carboxymethyllysine and *N*,*N*,*N*-trimethyl-5-aminovalerate) and 1 unknown metabolite were significantly reduced while the compound 1-methyl-5-imidazoleacetate was significantly increased (Supplementary Table S4). 

Murine plasma from RMC-f treatment showed changes in several amino acid metabolites involved in glutathione, methionine, histidine, tryptophan, and tyrosine metabolic pathways. There was increased 2-hydroxybutyrate, 3-methylhistidine and pipecolate and decreased *N*,*N*,*N*-trimethyl-5-aminovalerate, indoleacetate, methionine, and phenol sulfate levels (Supplementary Table S4). Forty-three lipid metabolites involved in a wide variety of fatty acid metabolic pathways were increased in the plasma of in mice receiving rice bran modified inoculum (Supplementary Table S4). Other notable changes included increased 7-ketodeoxycholate (a secondary bile acid) and decreased hippurate, salicylate, ferulic acid 4-sulfate, and 4-hydroxycinnamate in the plasma of in mice receiving rice bran modified inoculum (Supplementary Table S4). These decreased levels in plasma were notable for association with higher abundance for cancer protective actions in colon tissue. 

#### 3.5.2. Murine Metabolite Changes from Human Male FMT before and after Rice Bran Intervention

PCA of metabolite compositions of the proximal and distal colon tissue as well as plasma of mice receiving RMC-m and CMC-m are shown in Figure 6B. Differential abundance testing of metabolites revealed 52 metabolites increased and 65 decreased in proximal colon tissue; two metabolites increased, and 273 metabolites decreased in distal colon tissue; 60 metabolites increased and 31 decreased in plasma (Supplementary Table S4). 

Across both colon tissue locations, 3-ureidoisobutyrate was increased and a total of 46 metabolites including glutathione oxidized (GSSG), 3-methylhistidine, imidazole lactate, pipecolate, kynurenine, and quinolinate were decreased in mice receiving rice bran modified inoculum (Supplementary Table S4). Other metabolites that decreased were kynurenate and trigonelline in proximal colon tissue. Decreased ursocholate (a secondary bile acid) and reduced cysteine-glutathione disulfide in distal colon tissue of RMC-male transfaunated mice were also noted of cancer protective importance (Supplementary Table S4). 

For plasma metabolites, RMC-m showed increased taurodeoxycholate (a secondary bile acid), myristoylcarnitine and *N*,*N*,*N*-trimethyl-5-aminovalerate, and decreased methionine, indoleacrylate, 4-hydroxyhippurate, and hippurate (Supplementary Table S4).

**Figure 7:**
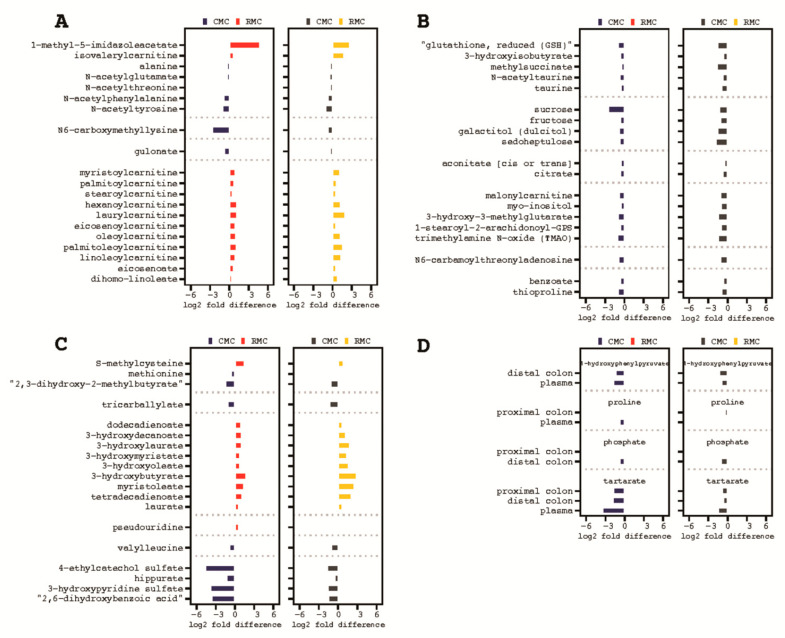
Differentially abundant metabolites in colon and plasma following control fecal microbial transplantation (CMC) and rice bran modified fecal microbial transplantation (RMC). (**A**), proximal colon. (**B**), distal colon. (**C**), plasma. (**D**), conserved metabolites across two or more sample types. Left panel represent the human female donor and right sides within each panel represent mice transfaunated with consortia from the human male donors.

Metabolites were analyzed for consistent increased or decreased abundance in mice receiving rice bran modified inoculum. Thirteen metabolites, including 1-methyl-5-imidazoleacetate, myristoylcarnitine and palmitoylcarnitine were consistently increased and ten metabolites including *N*6-carboxymethyllysine were consistently decreased in the proximal colon tissue (Table 1 and Figure 7A). 

The distal colon tissue of mice receiving rice bran modified inoculum displayed 25 consistently decreased metabolites including reduced glutathione (GSH), 3-hydroxyisobutyrate, myo-inositol, trimethylamine N-oxide (TMAO), benzoate, and thioproline (Table 1 and Figure 7B). Seventeen metabolites were consistently increased, and 14 metabolites were decreased in the plasma of these mice as well. Increased plasma metabolites included S-methylcysteine and 3-hydroxybutyrate and decreased metabolites included methionine, tricarballylate, and hippurate (Table 1 and Figure 7C). Metabolites consistently decreased in RMC transfaunated mice (across two or more sample types) included decreased phosphate in proximal and distal colon tissue, decreased proline in proximal colon tissue and plasma, and decreased 4-hydroxyphenylpyruvate in distal colon tissue and plasma. Interestingly, tartarate was the only metabolite with a conserved decrease across all three sample types (Table 1 and Figure 7D).

## 4. Discussion

This study of human donor stool microbiota transplantation before and after rice bran intake (CMC and RMC) into AOM-DSS treated mice demonstrated that RMC significantly reduced neoplastic lesions in colon when compared to colon tissue from CMC. The stool microbiota consortium from the female donor elicited greater reduction in murine colon tumor burden when compared to the consortium from the male donor, supporting that differences in microbial beta diversity were not strong indicators of FMT-mediated colon cancer protection. Furthermore, the metabolites identified in murine plasma, colon, cecum and feces following FMT indicated essential roles for metabolic functions by the gut microbiota. 

Notably, *Bacteroides fragilis*, *Enterococcus*, and *Parabacteroides distasonis* were enriched in the CMC-m inoculums that had more colon tumors in mice and were absent from the RMC-m inoculums that were protective against colon tumor formation. These taxa were shown to be enriched in the gut microbiota of CRC patients [48,49]. The depletion of *Parabacteroides distasonis* in mice transfaunated with RMC, which harbored fewer colonic lesions, is in contrast to a number of studies that have reported associations of *P. distasonis* with high tumor burdens [50], or pro-inflammatory activity increasing the severity of DSS-induced colitis [51]. However, at least one other study has reported anti-cancer mechanisms for *P. distasonis* [52]. Although direct comparisons of our study to these others are hindered by a number of key differences in experimental design [53,54], continued investigation of *P. distasonis* in CRC is warranted. 

To determine human stool microbiota transferability and to assess FMT goals for enrichment of selected taxa that are protective against colon cancer, we explored differences in microbial community composition along the murine gastrointestinal tract. Multiple sampling sites herein (e.g., cecum, proximal and distal colon, and feces collected over time) provided a strong snapshot of the gastrointestinal microbiome after three months that helped to elucidate site-specific microbial variations and to relationships reported in the literature. We found location-based differences between the cecum and proximal colon communities, while the proximal and distal colon locations harbored similar compositions in the mice. Consistent with our observations comparing the rice bran modified and control microbiota inoculums, there were enrichments of protective bacteria in both the cecum and the colon of mice receiving rice bran modified microbiota from the male and female donor inoculum.

Following observations in murine microbiota colonization by FMT in AOM-DSS mice, metabolite distinctions were also determined. Non-targeted metabolomics was also performed on murine colonic tissue and plasma (Supplementary Table S4). The distal colon tissue of mice receiving RMC displayed 25 consistently decreased metabolites including reduced glutathione (GSH), 3-hydroxyisobutyrate, myo-inositol, trimethylamine N-oxide (TMAO), benzoate, and thioproline (Table 1 and Figure 7B). Seventeen metabolites were consistently increased, and 14 metabolites were decreased in the plasma of mice receiving rice bran modified inoculum. Increased plasma metabolites included S-methylcysteine and 3-hydroxybutyrate and decreased metabolites included methionine, tricarballylate, and hippurate (Table 1 and Figure 7C). Metabolites consistently decreased in RMC mice (across two or more sample types) included decreased phosphate in proximal and distal colon tissue, decreased proline in proximal colon tissue and plasma, and decreased 4-hydroxyphenylpyruvate in distal colon tissue and plasma. Interestingly, tartarate was the only metabolite with a conserved decrease across all three murine biospecimens (Table 1 and Figure 7D). The distal colon tissue of RMC transplanted mice displayed 25 consistently decreased metabolites including reduced glutathione (GSH), 3-hydroxyisobutyrate, myo-inositol, and trimethylamine N-oxide (TMAO). The production of gut microbial modified metabolites alongside microbiota colonization after FMT is an exciting new avenue for identification of biomarkers associated with reduced inflammation that drives gastrointestinal diseases such as colon cancer. 

Two major findings to highlight for metabolites were the reduction of TMAO and tartrate in RMC mice as associations between these metabolites and development of CRC have been reported [28,30]. N6-carboxymethyllysine and 1 unknown metabolite were significantly reduced. We have already shown that N6-carboxymethlysine is decreased with rice bran consumption over time and increased in control diet consumers [24] and it is interesting that FMT alone seems to have the same effect on mice as humans receiving a rice bran enriched diet. This study also revealed candidate utility of *N*,*N*,*N*-trimethyl-5-aminovalerate to be considered for use as a dietary biomarker of rice bran in high risk colon cancer patients. The lower abundance of *N*,*N*,*N*-trimethyl-5-aminovalerate was also detected in murine plasma after FMT. Further investigation is warranted into the usefulness of other metabolites involved in a wide variety of fatty acid metabolic pathways that were either increased or decreased. Three metabolites with previously reported anti-cancer activity that were increased in RMC transplanted mice include (myristoylcarnitine) [55] and palmitoylcarnitine [55], both increased in the proximal colon, and *S*-methylcysteine [56] increased in the plasma. 

The study design and novel approach applied herein have resulted in findings of importance to future cancer control and prevention strategies related to diet and FMT. We also acknowledge a number of limitations to this investigation. In particular, the sequencing utilized herein was limited to bacteria and does not discern between living or dead cells [57]. We did not capture the metabolic activities at the transcript or protein level for the bacterial communities that showed differential abundance for integration with metabolite production. The selection of microbial communities with protection in the murine model may differ from human FMT utility due to a series of metabolic differences between mice and humans (e.g., the primary role for cecal fermentation in mice). We also appreciate larger differences between the proximal and distal colon metabolism of humans [58]. Comprehensive multi-omics analysis of the microbial inoculums used for FMT and the host system will further advance the amplicon/or marker gene-based studies [59]. Moreover, complementary approaches with other dietary-whole grain interventions prior to fecal transplantation are warranted to determine the specificity of this response to rice bran when compared to other whole grain cereals.

## 5. Conclusions

Results from this study show that human rice bran diet-modified stool microbial consortia (RMC) reduced chemically-induced murine colon carcinogenesis. This represents a novel approach to understanding the integrated role of dietary modification to microbial communities and metabolic functions of microbiota after fecal transplantation. Dietary intake of 30 g/day of rice bran for 28 days by CRC survivors was sufficient to produce gut microbial communities that differentially colonized germ-free mice, altered host and gut microbial metabolism, and conferred protection from AOM/DSS-induced colon carcinogenesis. This is the first study to show that the beneficial effects of consuming rice bran are transferable via FMT. Furthermore, we identified a number of microbiota and several specific metabolites that were associated with these colon health benefits. We conclude the potential benefits for precision nutrition with rice bran to impact human FMT and as a novel strategy for colon cancer prevention. 

## Figures and Tables

**Table 1:** Selected blood and intestinal metabolites and microbial taxa with relationships to the cancer protective outcomes following fecal microbiota transplantation (FMT) with rice bran modified microbiota (RMC) in AOM-DSS treated mice.

**Metabolites Increased in Rice Bran Modified Microbial Consortia (RMC)**	
**Metabolite**	**Sample Type**
Isovalerylcarnitine	Proximal Colon
1-methyl-5-imidazoleacetate	Proximal Colon
Dihomo-linoleate	Proximal Colon
Eicosenoate	Proximal Colon
Linoleoylcarnitine	Proximal Colon
Palmitoleoylcarnitine	Proximal Colon
Oleoylcarnitine	Proximal Colon
Eicosenoylcarnitine	Proximal Colon
Laurylcarnitine	Proximal Colon
Hexanoylcarnitine	Proximal Colon
Stearoylcarnitine	Proximal Colon
Palmitoylcarnitine	Proximal Colon
Myristoylcarnitine	Proximal Colon
*S*-methylcysteine	Plasma
Laurate (12:0)	Plasma
Tetradecadienoate (14:2)	Plasma
Myristoleate (14:1n5)	Plasma
3-hydroxybutyrate (BHBA)	Plasma
3-hydroxyoleate	Plasma
3-hydroxymyristate	Plasma
3-hydroxylaurate	Plasma
3-hydroxydecanoate	Plasma
Dodecadienoate (12:2)	Plasma
Pseudouridine	Plasma
Unknown 12101	Plasma
Unknown 24623	Plasma
Unknown 23678	Plasma
Unknown 21353	Plasma
Unknown 18921	Plasma
Unknown 17335	Plasma
**Metabolites Decreased in Rice Bran Modified Microbial Consortia (RMC)**	
**Metabolite**	**Sample Type**
*N*-acetyltyrosine	Proximal Colon
*N*-acetylphenylalanine	Proximal Colon
*N*-acetylthreonine	Proximal Colon
*N*-acetylglutamate	Proximal Colon
aAanine	Proximal Colon
*N*6-carboxymethyllysine	Proximal Colon
Gulonate	Proximal Colon
Taurine	Distal Colon
*N*-acetyltaurine	Distal Colon
Methylsuccinate	Distal Colon
Glutathione, reduced (gsh)	Distal Colon
Sedoheptulose	Distal Colon
Galactitol (Dulcitol)	Distal Colon
Fructose	Distal Colon
Sucrose	Distal Colon
Citrate	Distal Colon
Aconitate [cis or trans]	Distal Colon
Trimethylamine N-oxide (TMAO)	Distal Colon
1-stearoyl-2-arachidonoyl-GPS (18:0/20:4)	Distal Colon
3-hydroxy-3-methylglutarate	Distal Colon
Myo-inositol	Distal Colon
Malonylcarnitine	Distal Colon
*N*6-carbamoylthreonyladenosine	Distal Colon
Thioproline	Distal Colon
Benzoate	Distal Colon
Unknown 24952	Distal Colon
Unknown 24431	Distal Colon
Unknown 24027	Distal Colon
2,3-dihydroxy-2-methylbutyrate	Plasma
Methionine	Plasma
Tricarballylate	Plasma
Calylleucine	Plasma
2,6-dihydroxybenzoic acid	Plasma
3-hydroxypyridine sulfate	Plasma
Hippurate	Plasma
4-ethylcatechol sulfate	Plasma
Unknown 12221	Plasma
Unknown 11850	Plasma
Unknown 07765	Plasma
Phosphate	Proximal Colon and Distal Colon
Proline	Proximal Colon and Plasma
4-hydroxyphenylpyruvate	Distal Colon and Plasma
Tartarate	Proximal Colon, Distal Colon, and Plasma
**Taxa Depleted in Rice Bran Modified Microbial Consortia (RMC)**	
**Assignment**	**Sample Type**
*Parabacteroides distasonis*	Cecum, Human Stool Inoculum
Tannerellaceae	Cecum, Proximal Colon

## Data Availability

Sequence data supporting the conclusions of this manuscript are available under NCBI BioProject PRJNA630498. Materials needed to reproduce the analysis may be found on this project’s GitHub repository located at github.com/ kdprkr/TransFaunation.

## Supplementary Materials

The following are available online at https://www.mdpi.com/2227-9059/9/2/144/s1, Figure S1: Tumor outcomes by colon location and effects of murine sex on tumor outcomes. Figure S2: Proportions of taxa in human stool inoculums and murine fecal, cecum, and colon samples having previous associations with colorectal cancer or colon tumor burden, Table S1: Results of statistical comparisons for lesion outcomes and microbial community beta diversity, Table S2: Differentially abundant taxa and amplicon sequence variants in human stool inoculums and murine samples, Table S3: List of taxa previously associated with colorectal cancer or colon tumor burden, Table S4: Differentially abundant metabolites in murine samples. Full details regarding metataxonomics protocols and analysis and sample processing and metabolite identification can be found in the Supplementary Methods.
