# Supplementary material for: Dietary Rice Bran-Modified Human Gut Microbial Consortia Confers Protection against Colon Carcinogenesis Following Fecal Transfaunation
Source: Biomedicines. 2021 Feb 3;9(2):144. doi: 10.3390/biomedicines9020144 (PMC7913285; doi:10.3390/biomedicines9020144)
Supplement: Supplementary file 1 [file biomedicines-09-00144-s001.zip › sup/Supplementary_Figure_S1.pdf]

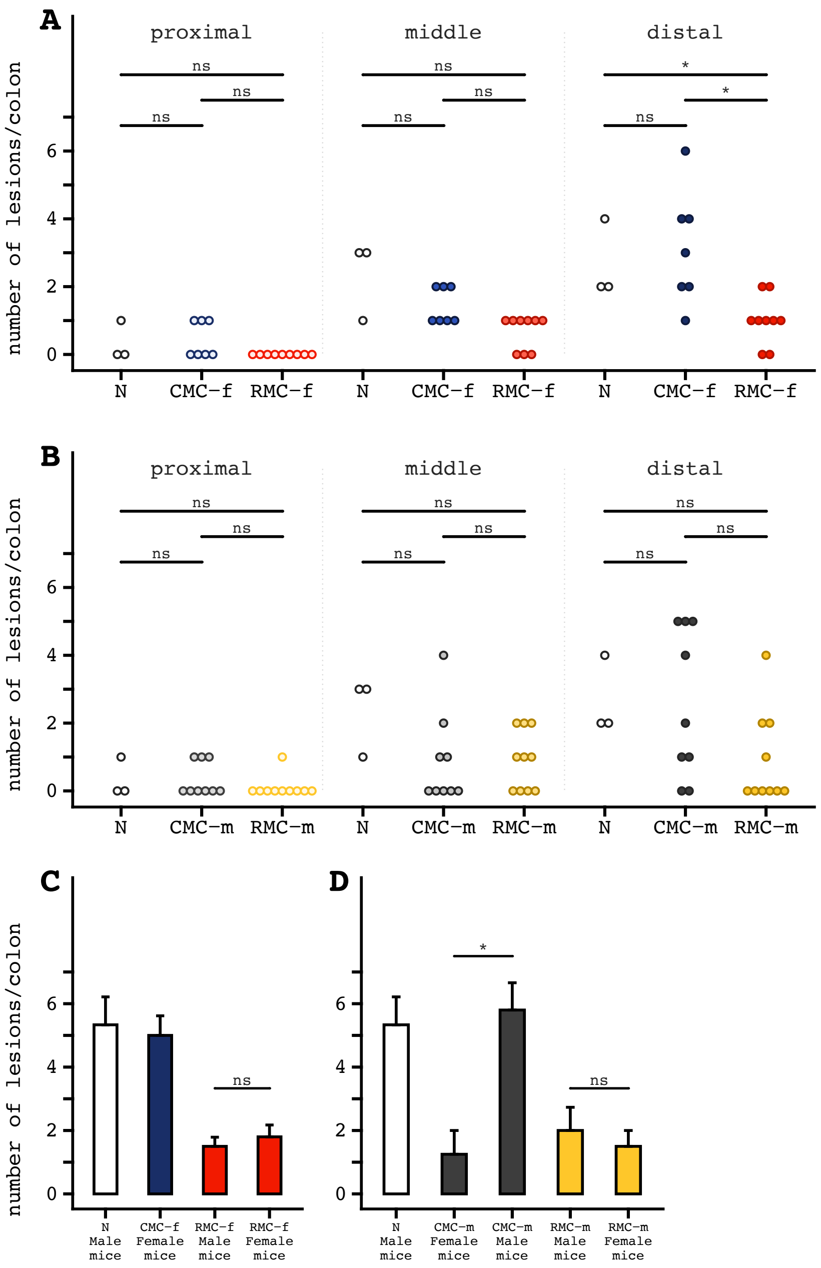

Supplementary Figure S1

Tumor outcomes by colon location (A-B) and effects of murine sex on tumor outcomes (C-D).

A, Number of neoplastic lesions in total proximal, middle, and distal regions of colon tissue for mice receiving no microbial consortia (N) when compared to control microbial consortia (CMC-f) and rice bran modified microbial consortia (RMC-f) from human female. **B,** Number of neoplastic lesions in total proximal, middle, and distal regions of colon tissue for mice receiving no microbial consortia (N) when compared to control microbial consortia (CMC-m) and rice bran modified microbial consortia (RMC-m) from human male. **C-D,** Number of neoplastic lesions in entire colon tissue comparing the sex of the mouse within individual recipient groups. Error bars represent standard error of the mean; (**) indicates Benjamani-Hochberg adjusted P-value < 0.01; (*) indicates Benjamani-Hochberg adjusted P-value < 0.05; (ns) indicates Benjamani-Hochberg adjusted P-value > 0.05.
