# Supplementary material for: Dietary Rice Bran-Modified Human Gut Microbial Consortia Confers Protection against Colon Carcinogenesis Following Fecal Transfaunation
Source: Biomedicines. 2021 Feb 3;9(2):144. doi: 10.3390/biomedicines9020144 (PMC7913285; doi:10.3390/biomedicines9020144)
Supplement: Supplementary file 1 [file biomedicines-09-00144-s001.zip › sup/Supplementary_Figure_S2.pdf]

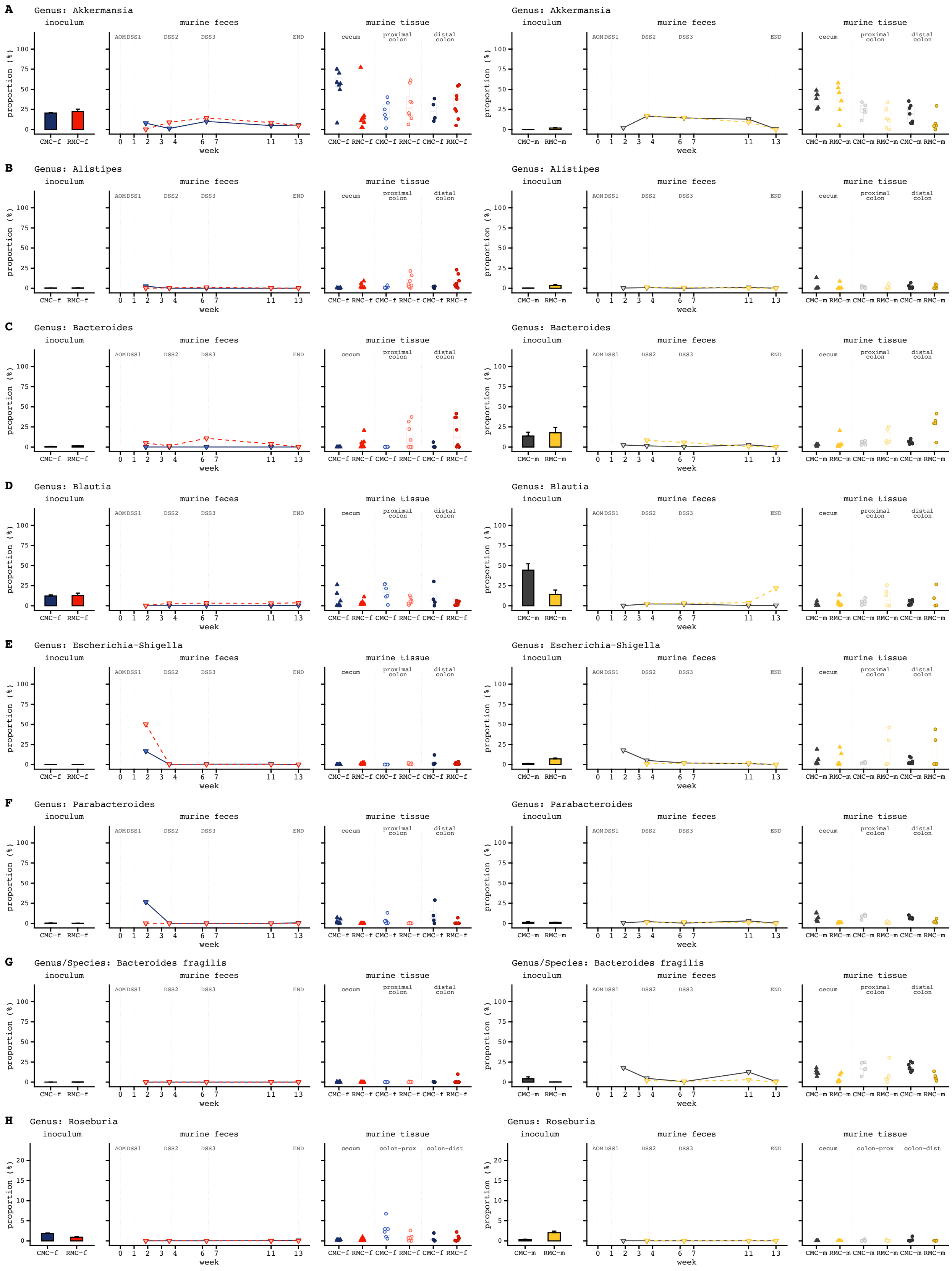

Supplementary Figure S2 - (continued on following page)

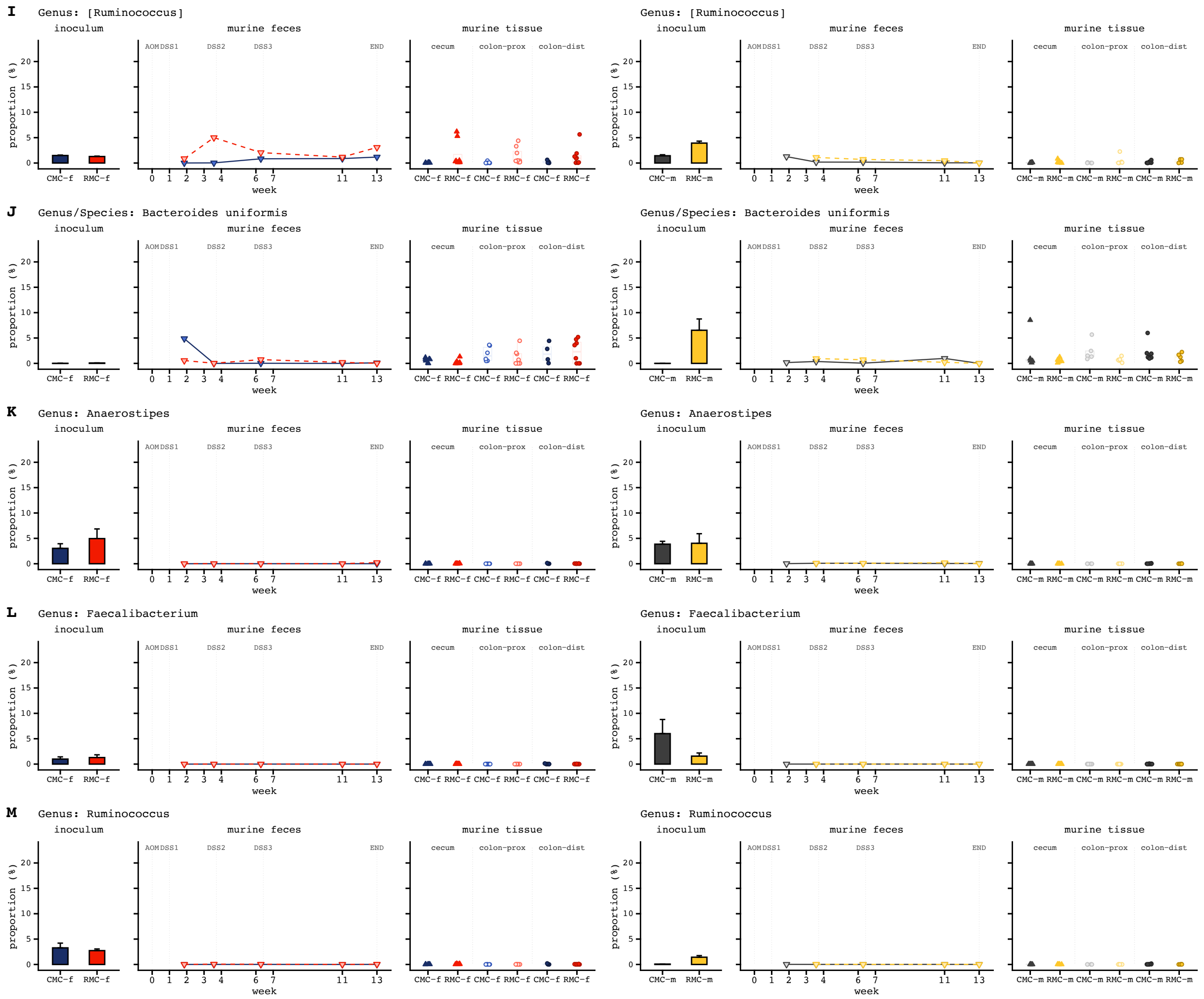

Supplementary Figure S2

Proportions of taxa in human stool inoculums and murine fecal, cecum, and colon samples having previous associations with colorectal cancer or colon tumors.

A-M, proportions for stool inoculums and samples from mice colonized with consortia derived from the human female (left) and human males (right). A-G, proportions for taxa reported as enriched in colorectal cancer or enriched in groups with high tumor counts or positively correlated with increased tumor burden or enriched. H, taxon with mixed reports as depleted in colorectal cancer or enriched in groups with high tumor counts. I-M, taxa reported as depleted in colorectal cancer. C, Bacteroides proportions depicted do not include Bacteroides fragilis or Bacteroides uniformis abundances. I, brackets [ ] indicate contested assignment. The y-axis is identical across plots within a given panel and is set at 100% for A-G and 20% for H-M. Taxonomy was collapsed at the genus or species level to combine proportions for identical assignments prior to visualization. Taxonomy was assigned using the SILVA 132 marker gene database.
